# The Effects of Combined Therapy With Metformin and Hydroxypropyl-β-Cyclodextrin in a Mouse Model of Niemann-Pick Disease Type C1

**DOI:** 10.3389/fphar.2021.825425

**Published:** 2022-01-14

**Authors:** Jiang Du, Xinlei Liu, Yan Zhang, Xiaojing Han, Chunya Ma, Yanli Liu, Lihong Guan, Liang Qiao, Juntang Lin

**Affiliations:** ^1^ College of Medical Engineering, Xinxiang Medical University, Xinxiang, China; ^2^ Stem Cell and Biotherapy Engineering Research Center of Henan, Xinxiang Medical University, Xinxiang, China; ^3^ College of Life Science and Technology, Xinxiang Medical University, Xinxiang, China

**Keywords:** NPC1 disease, HPβCD, metformin, cholesterol accumulation, combined therapy

## Abstract

Niemann–Pick disease type C1 (NPC1) is a neurodegenerative disorder characterized by lysosomal storage of free cholesterol. 2-Hydroxypropyl-β-cyclodextrin (HPβCD) is a cyclic oligosaccharide derivative that is being developed to treat NPC1. Recently, metformin was reported to be beneficial in various neurodegenerative diseases, such as Alzheimer’s and Huntington’s diseases. In this study, we examined the effects of combined treatment with HPβCD and metformin on *Npc1* ^*−/−*^ mice. Unfortunately, body weight and survival rates showed that cotreatment with metformin did not extend survival time and increase the body weight of HPβCD-treated *Npc1* ^*−/−*^ mice. However, cotreatment with metformin reduced inflammatory response and inhibited the proinflammatory cytokine release in the brain, liver and spleen of HPβCD-treated *Npc1* ^*−/−*^ mice. Furthermore, metformin did not reduce the free cholesterol levels in *Npc1* ^*−/−*^ brain tissue or fibroblasts. In conclusion, our results demonstrate that metformin does not show beneficial effects on body weight or survival time but reduced the inflammatory response in a mouse model of NPC1 when combined with HPβCD.

## Introduction

NPC1 is a rare, neurodegenerative, inherited recessive disease caused by mutations in the *Npc1* or *Npc2* gene (Vanier, 2010). These mutations affect the intracellular trafficking of cholesterol and other lipids, which leads to the progressive accumulation of unesterified cholesterol in the CNS and other organs (Vanier, 2015). Unfortunately, there are few well-established pharmacological approaches to treat NPC. In preclinical studies, 2-hydroxypropyl-β-cyclodextrins (HPβCD) significantly delayed cerebellar Purkinje cell loss, slowed the progression of neurological manifestations, and increased lifespans in mouse and cat models of NPC1 (Peake and Vance, 2012; Vite et al., 2015). Recently, patients with NPC1 who were treated with intrathecal HPβCD exhibited slowed disease progression and an acceptable safety profile in an open-label, dose-escalation phase 1-2a study (Ory et al., 2017). Although the mechanism by which HPβCD affects NPC1 is not understood, this treatment has been shown to be effective in reversing the intracellular accumulation of cholesterol and associated lipids in neuronal cell lines.

Metformin is the most frequently used oral antidiabetic drug. Metformin has been indicated to decrease plasma glucose levels and exert anti-inflammatory, antiapoptotic and antioxidative effects through several mechanisms (Mahmood et al., 2013). Recently, metformin has proven protective in a wide variety of animal models of neurodegenerative diseases, such as Alzheimer’s disease, Parkinson’s disease, and Huntington’s disease (Cardoso and Moreira, 2020). Most neurodegenerative diseases share pathological mechanisms of neuroinflammation and cell damage that occur through parallel stress pathways in diabetes (Gantois et al., 2019). Mechanistically, metformin can enhance neuronal bioenergetics, promote nerve repair and reduce toxic protein aggregates in neurological diseases through the activation of AMPK and suppression of the mammalian target of rapamycin (mTOR) pathway (Demaré et al., 2021). The clinical features of NPC1 show severe neuroinflammation. Similar to other neurodegenerative diseases, activation of the innate immune system occurs in the brain, resulting in neuroinflammation (Cologna et al., 2014). Some studies have reported that *Npc1* deficiency or cholesterol trafficking inhibition leads to synergistic inhibition of mTOR signaling (Head et al., 2017; de la Roche et al., 2018). Methyl-β-cyclodextrin, a potent analog of HPβCD, restores impaired autophagic flux in Npc1-deficient cells through the activation of AMPK (Dai et al., 2017). These studies identify AMPK/mTOR as an attractive target for the development of drugs to treat NPC1. Because AMPK is the primary target of metformin, we hypothesized that metformin may have a beneficial effect on NPC1 in a mouse model. However, no study has reported the role of metformin in NPC1.

In this study, we examined the effects of combined treatment with HPβCD and metformin on *Npc1* ^*−/−*^ mice. Compared with the treatment of HPβCD alone, cotreatment with metformin did not extend life span and increase body weight in HPβCD-treated *Npc1* ^*−/−*^ mice. However, combination therapies have the potential to reduce inflammatory response and inhibit the proinflammatory cytokine release in the brain, liver and spleen of *Npc1* ^*−/−*^ mice. Further, metformin did not reduce the free cholesterol levels in *Npc1* ^*−/−*^ brain tissue or fibroblasts. In summary, our results demonstrate that metformin can reduce the inflammatory response but not improve the lifespan in a mouse model of NPC1 when combined with HPβCD.

## Materials and Methods

### Animal Experiments

Heterozygous *Npc1* mutant mice (BALB/cNctr-*Npc1* ^m1N^/J) were obtained from Jackson Laboratory (United States) and bred to generate homozygous Npc1 mutants (*Npc1* ^*−/−*^). HPβCD (Sigma) and metformin (Sigma) were dissolved in PBS. A total of 53 age-matched (P20) *Npc1* ^*−/−*^ mice were divided into the following four groups: 1) the control group was treated with PBS (20 μL/g) (*n* = 15; nine males and six females); 2) the HPβCD group was treated with 2000 mg/kg HPβCD every other day (n = 13; seven males and six females); 3) the metformin group was treated with 100 mg/kg/day metformin (*n* = 12; seven males and five females); and 4) the metformin and HPβCD group was treated with 2000 mg/kg HPβCD every other day and 100 mg/kg/day metformin (*n* = 13; seven males and six females). The dose of HPβCD or metformin solution was 20 μL/g in all of the drug-treated groups. All mutant mice received drugs at 20 days of age by intraperitoneal injection. Body weight was measured every other day until reaching the late humane end-point (loss of 1 g body weight within 24 8) as previously described (Williams et al., 2014). All mice were bred and housed under nonsterile conditions, with food and water available ad libitum. The Guidelines of the Animal Care Committee of Xinxiang Medical University were followed when carrying out the *in vivo* experiments.

### Immunofluorescence Staining

Immunofluorescence staining are used to identify phagocytic macrophage (CD68^+^) infiltration or microglia expression (Iba1). Briefly, the liver sections were fixed in 4% paraformaldehyde and permeabilized with 0.1% Triton X-100. Then, the slides were incubated with the primary CD68 or Iba1 antibody overnight at 4°C. Then the samples were incubated in PBS for 1 h at room temperature with secondary antibodies [goat anti-rabbit Alexa Fluor 594 (1:200) or goat anti-rabbit Alexa Fluor 488 (1:200)] after washing three times for 10 min each time. The nuclei were counterstained with DAPI (Sigma). Finally, the immunofluorescence staining was analysed using a laser-scanning confocal microscope (Leica) and quantitatively determined by ImageJ.

### qPCR Assay

For qPCR assays, reverse transcription total RNA was isolated using TRIzol reagent (#W9514, Tiangen) from either tissue samples or cultured cells. Total RNA was obtained and then reverse-transcribed was performed with 2 μg total RNA using the Reverse Transcription Kit (#PIA279, Promega) following the manufacturer’s instructions. qRT-PCR experiments were carried out by an CFX96 real-time PCR system (Bio-Rad, C1000) using SYBR green real-time PCR master mix (G891, abm). The mRNA expression levels of the target genes were normalized to Gapdh or actin expression. The primer pairs used in this study are listed in Table 1.

**TABLE 1:** Primers for qPCR.

Gene	Sequence
Mouse *cd68*	Forward: GGC​GGT​GGA​ATA​CAA​TGT​GTC​C
	Reverse: AGC​AGG​TCA​AGG​TGA​ACA​GCT​G
Mouse *Tnfα*	Forward: AGG​GTC​TGG​GCC​ATA​GAA​CT
	Reverse: CCA​CCA​CGC​TCT​TCT​GTC​TAC
Mouse *il6*	Forward: TCC​ATC​CAG​TTG​CCT​TCT​TG
	Reverse: AAG​CCT​CCG​ACT​TGT​GAA​GTG
Mouse *il1β*	Forward: GGT​CAA​AGG​TTT​GGA​AGC​AG
	Reverse: TGT​GAA​ATG​CCA​CCT​TTT​GA
Mouse *GAPDH*	Forward: GTT​GTC​TCC​TGC​GAC​TTC​A
	Reverse: GCC​CCT​CCT​GTT​ATT​ATG​G

### Filipin Staining

WT and *Npc1* ^*−/−*^ fibroblasts were isolated from the tails of WT and *Npc1* ^*−/−*^ mice. These cells were cultured in Dulbecco’s modified Eagle’s medium (DMEM) (HyClone) supplemented with 10% fetal bovine serum (FBS) (Gibco), 100 μg/ml penicillin and streptomycin at 37°C and 5% CO2. Normal and NPC1 fibroblasts (5 × 10^3^ cells) were seeded in 24-well plates and incubated overnight. The cells were treated with HPβCD (0.01, 0.1, 1, and 10 mM) or/and 10 mM metformin for 24 h. Intracellular cholesterol was visualized with Filipin III staining (Sigma) as described in a previous report (Tamura and Yui, 2018).

For visualization of unesterified cholesterol in brain tissue, the brain sections were fixed in 4% paraformaldehyde for 15 min, washed 3 × 5 min in PBS at room temperature, and incubated with 0.1 mg/ml filipin for 1 h. After three washing steps with PBS, the slides were analysed using a laser-scanning confocal microscope (Leica).

### Statistical Analysis

All experiments were repeated at least three times independently. Statistical analyses were performed using GraphPad Prism version 6 (GraphPad Software). Statistical comparisons between groups were performed using one-way ANOVAs followed by the Tukey test or Dunnett’s test. Differences were considered significant at the level *p* < 0.05 (*p* values <0.05 were considered significant, *p < 0.05, **p < 0.005, ***p < 0.001). The data are presented as the arithmetic mean ± standard error of the mean (SEM).

## Results

### Effects of HPβCD And/Or Metformin on Survival Times and Changes in Body Weight in Npc1^−/−^ Mice

**FIGURE 1:**
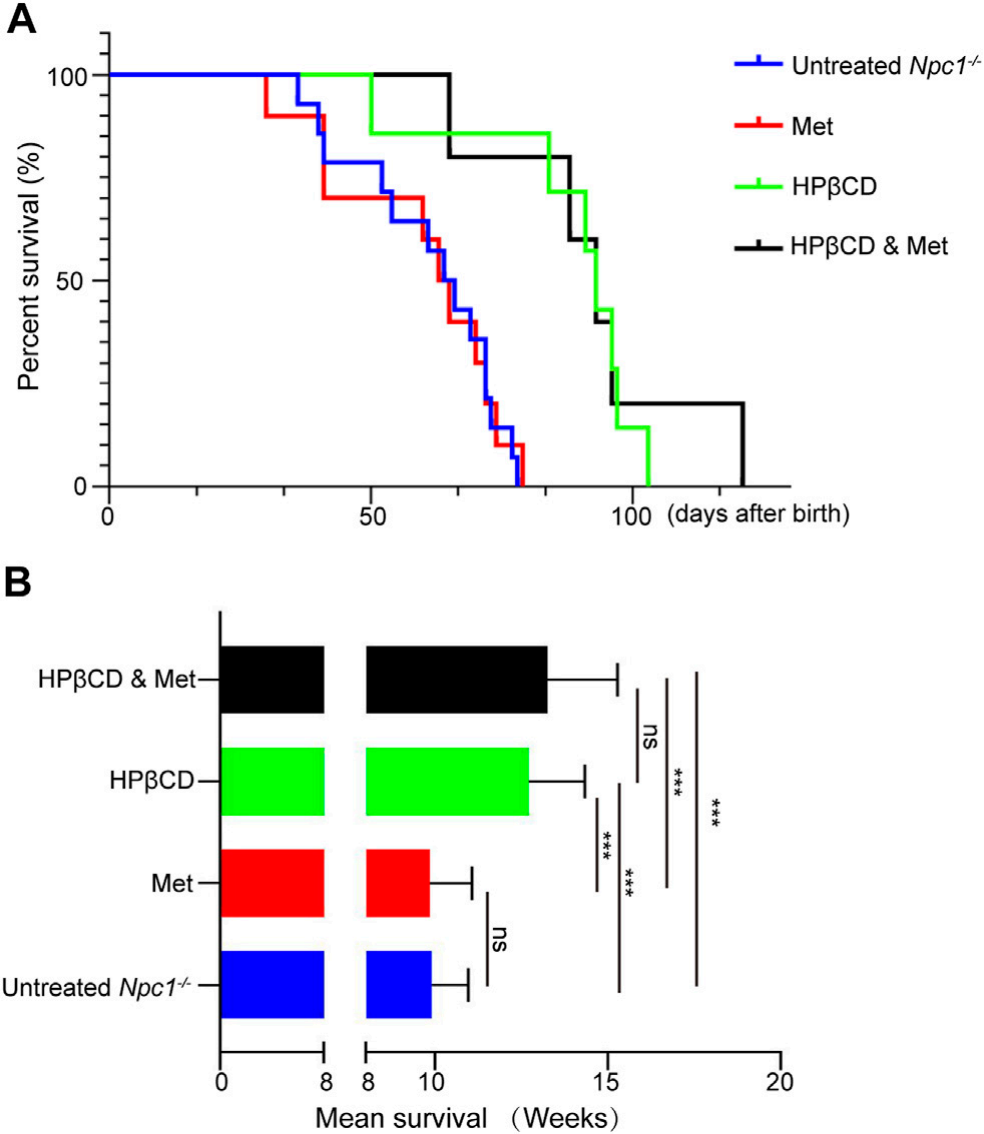
Effect of combination therapies on survival in *Npc1* ^*−/−*^ mice. Survival was evaluated applying a late humane end point and is presented as Kaplan–Meier survival plots **(A)** and mean survival **(B)**. Statistical analysis is based on one-way ANOVA followed by Tukey test, ns represents not signaficant, ***p < 0.001. All data are provided as means ± SEM.

Recent reports have shown that treatment with HPβCD delays clinical disease onset, reduces intraneuronal storage and secondary markers of neurodegeneration, and significantly increases the lifespans of *Npc1* ^*−/−*^ mice (Davidson et al., 2009). To confirm whether metformin is an effective treatment for NPC1 when combined with HPβCD, heterozygous *Npc1* ^*−/−*^ mice were bred to generate *Npc1* ^*−/−*^ mice. *Npc1* ^*−/−*^ mice were treated with PBS, monotherapies and combined therapy. The control *Npc1* ^*−/−*^ mouse model had an acute clinical course, and the mice died by 8–11 weeks of age, with a mean survival of 10 weeks. The mean lifespan did not show a clear change in response to monotherapy treatment with metformin. As previously reported, HPβCD extended the lifespan in the *Npc1* ^*−/−*^ mouse model and exerted additive benefits, with a 26% increase in lifespan relative to that of the control group. Combining metformin and HPβCD had a survival benefit, extending life expectancy (mean of 13.5 weeks), and inducing a 30% increase in lifespan compared to that of control mice. However, the combined treatment did not extend life expectancy compared to that of the HPβCD-treated mice (Figures 1A,B). Taken together, these results suggest that metformin does not prolong survival time in a mouse model of NPC1 when combined with HPβCD.

**FIGURE 2:**
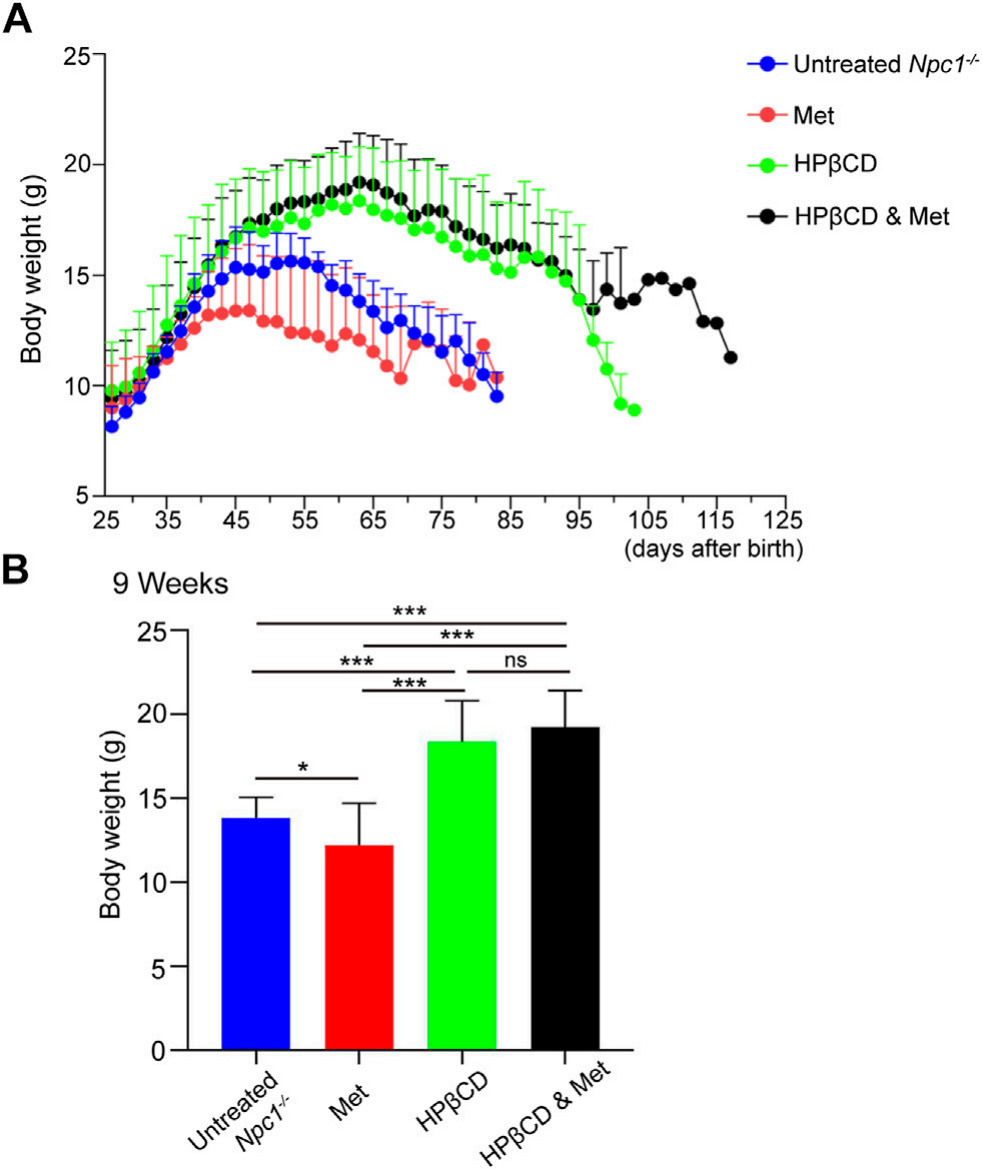
Effect of test drugs on body weight of in *Npc1* ^*−/−*^ mice. The mean weight for each treatment group is plotted against age **(A)**. The maintenance of weight in each treatment group is shown as the mean weight for each group at 9 weeks of age in **(B)**. Statistical analysis is based on one-way ANOVA followed by Tukey test, ns represents not significant, *p < 0.05, ***p < 0.001. All data are provided as means ± SEM.

Another indicator of the therapeutic effect on *Npc1* ^*−/−*^ mice is the maintenance of body weight (Davidson et al., 2009). Naturally, the body weights of *Npc1* ^*−/−*^ mice entered the stationary phase when the mice reached 4–5 weeks of age and began to decline progressively at 5–6 weeks. Previous studies have shown that weight loss is associated with metformin in both diabetic and nondiabetic individuals (Ouyang et al., 2020). In our study, metformin treatment slightly decreased the body weights of *Npc1−/−* mice at 5 weeks of age compared to those of control mice. The average body weight was significantly improved and decreased at 7–8 weeks of age in the HPβCD-treated group. However, combining metformin and HPβCD did not influence the body weights of *Npc1−/−* mice compared to HPβCD treatment alone. In 13 combination treatment mice, only one mouse survived to nearly 130 days and maintained a stable weight index at the later stage of life (Figures 2A,B). Despite individual differences, the results show that metformin does not change the body weights obviously of *Npc1−/−* mice when combined with HPβCD.

### Effects of HPβCD And/Or Metformin on Brain Inflammation in Npc1^−/−^ Mice

**FIGURE 3:**
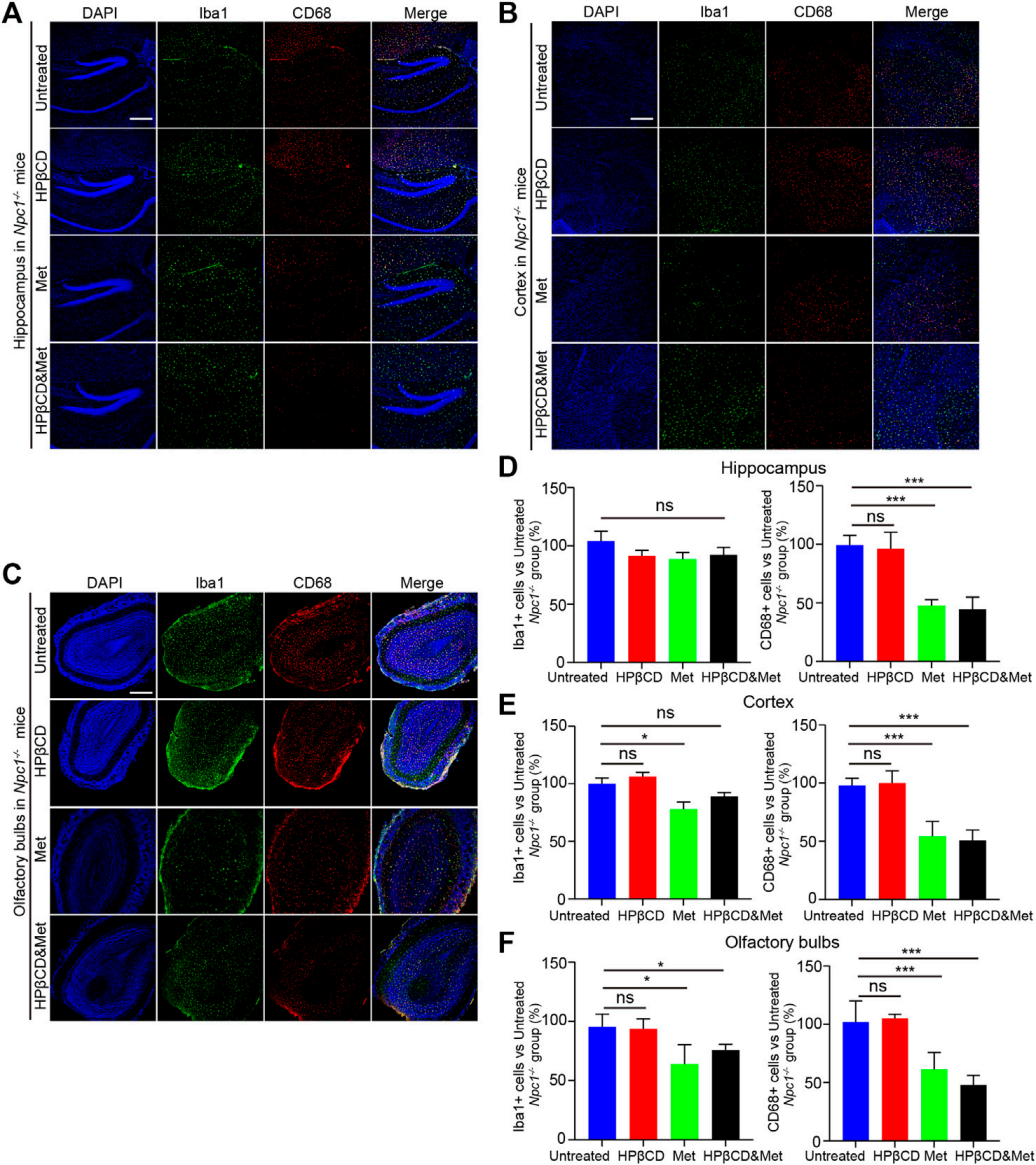
Effects of test drugs on Iba1 and CD68 expression in *Npc1* ^*−/−*^ brain. Representative images of Iba1 and CD68 expression in the hippocampus **(A)**, cerebral cortex **(B)** and olfactory bulb **(C)** in the indicated groups (*n* = 4) (scale bar: 100 μm). Quantitative analysis of Iba1 and CD68 positive cells in the hippocampus **(D)**, cerebral cortex **(E)** and olfactory bulb **(F)** using ImageJ. Significant differences were detected by one-way ANOVA followed by Dunnett’s test, ns represents not significant, *p < 0.05, ***p < 0.001. versus untreated group.

Inflammation in the brain is a hallmark of many neurodegenerative diseases, irrespective of the underlying cause. It has been reported that metformin has anti-inflammatory effects in many diseases. To test whether the treatments had any impact on brain inflammation, *Npc1* ^*−/−*^ mice were treated with metformin, HPβCD, metformin and HPβCD or fed standard chow as a control. The animals were sacrificed at 8 weeks of age. Subsequently, the Iba1 and CD68 expression in the hippocampus (HC), cerebral cortex (CC) and olfactory bulb (OB) were analysed as a measure of brain inflammation. The HPβCD treated *Npc1* ^*−/−*^ mice showed no significant difference on Iba1^+^ or CD68^+^ cells compared to untreated *Npc1* ^*−/−*^ animals. However, the metformin or combined drugs treated *Npc1* ^*−/−*^ animals presented with significantly less CD68^+^ cells and slightly less Iba1^+^ cells in all areas of the HC, CC and OB (Figures 3A–F). Furthermore, we therefore used quantitative PCR to study the expression profile of proinflammatory cytokine as neuroinflammatory factors in the brain of *Npc1* ^*−/−*^ mice at 8 weeks of age. The results showed the transcript level of *cd68* was decreased in the metformin or combined drugs treated *Npc1* ^*−/−*^ brain compared to untreated or HPβCD treated *Npc1* ^*−/−*^ animals. Meanwhile, we found the metformin or combined drugs can inhibit the proinflammatory cytokine (*Tnfα, il1β* and *il6*) release in the brain (Figures 5A–D). In summary, the combined therapy of metformin and HPβCD has a certain protective effect on NPC1 brain inflammation.

### Effects of HPβCD and/or Metformin on Hepatosplenic Inflammation in Npc1^−/−^ Mice

**FIGURE 4:**
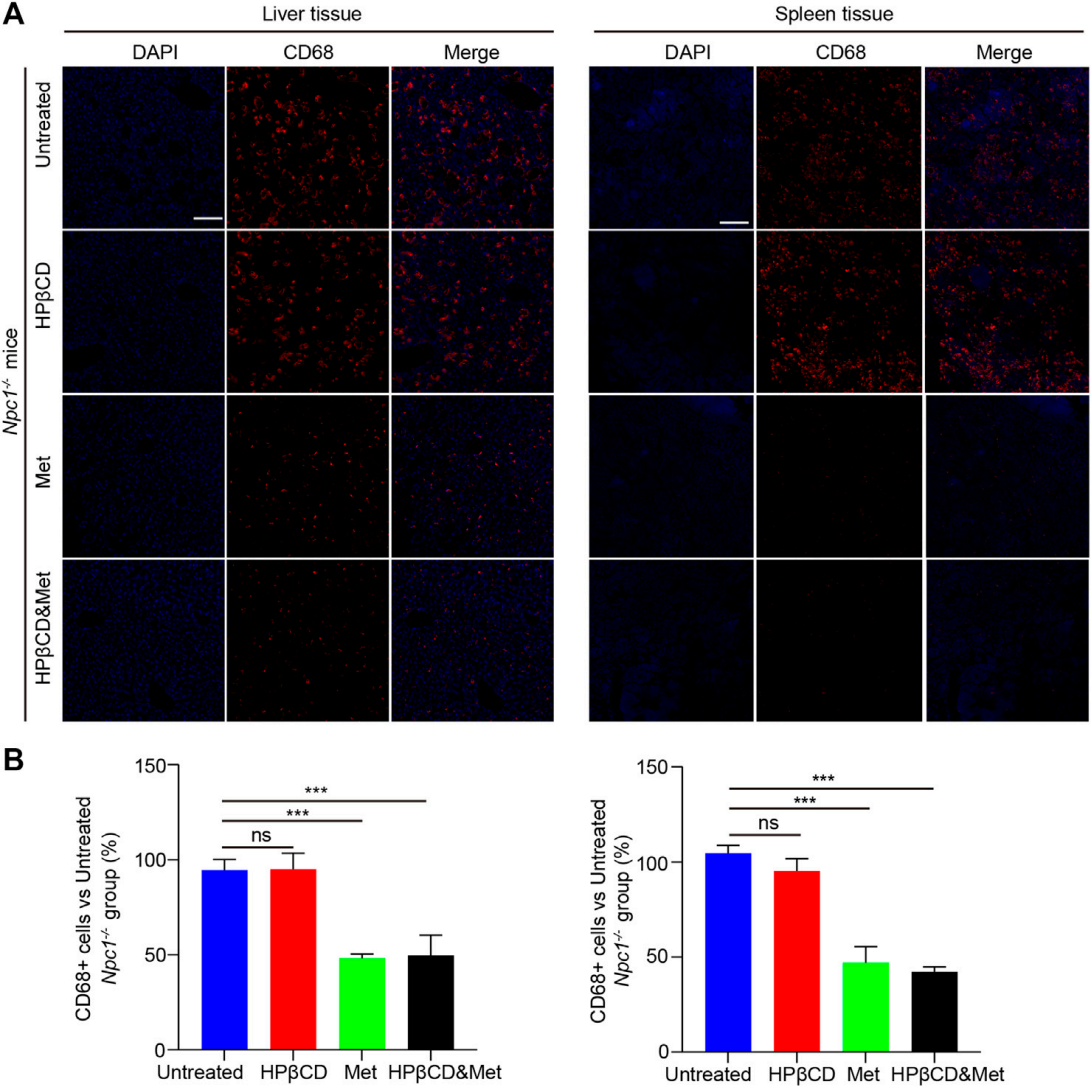
Effects of test drugs on CD68 expression in *Npc1* ^*−/−*^ liver and spleen. Representative images of Iba1 and CD68 expression in the liver (left) and spleen (right) in the indicated groups **(A)** (*n* = 4) (scale bar: 100 μm). Quantitative analysis of Iba1 and CD68 positive cells using Image J **(B)**. Significant differences were detected by one-way ANOVA followed by Dunnett’s test, ns represents not significant, ***p < 0.001. versus untreated group.

**FIGURE 5:**
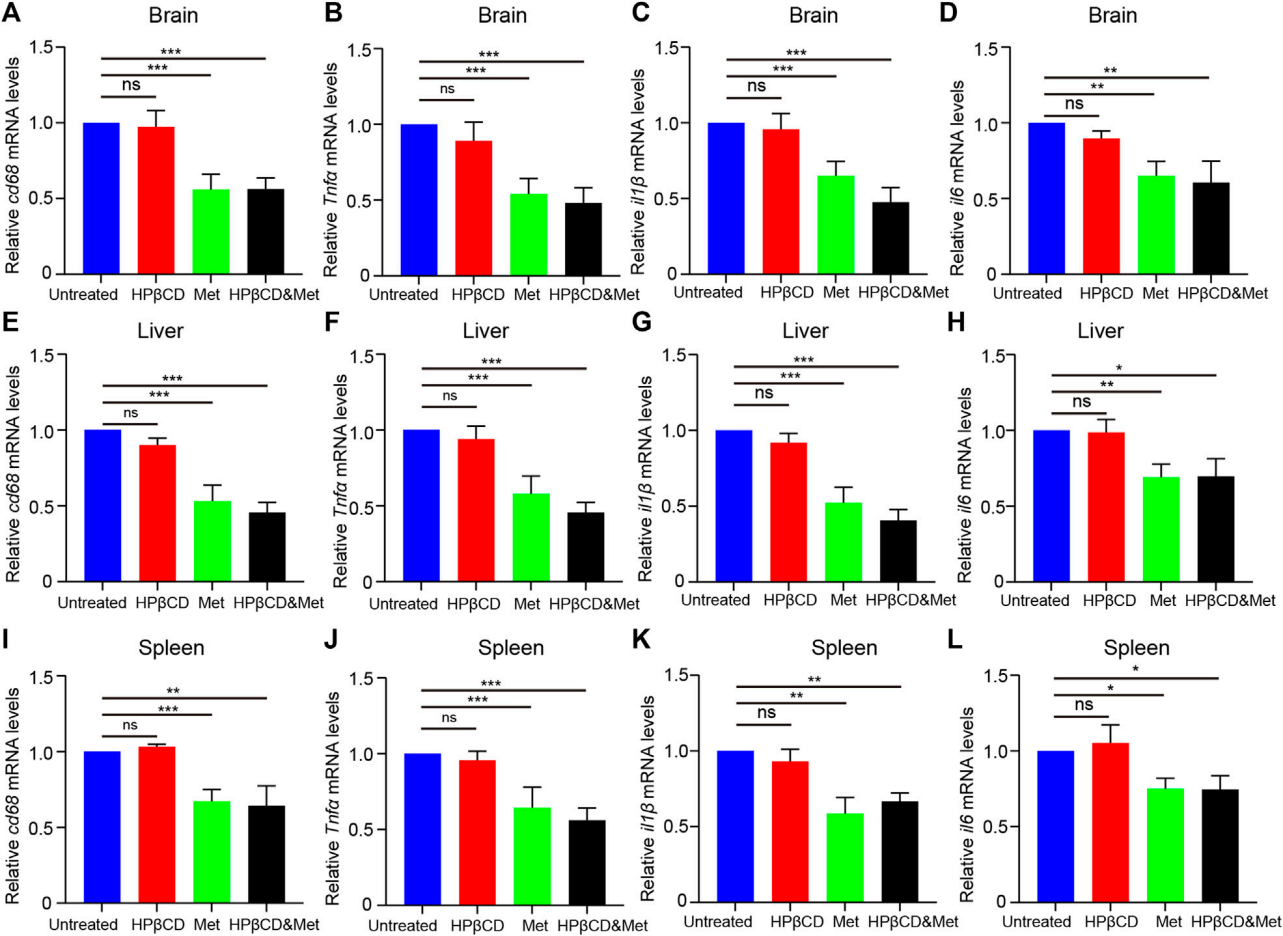
Effects of test drugs on proinflammatory cytokine release in *Npc1* ^*−/−*^ brain, liver and spleen. Quantitative PCR to study the expression profile of *cd68, Tnfα, il1β* and *il6* at 8 weeks of age in *Npc1* ^*−/−*^ brain **(A–D)**, liver **(E–H)** and spleen **(I–L)** (*n* = 4). Significant differences were detected by one-way ANOVA followed by Dunnett’s test, ns represents not significant, *p < 0.05, **p < 0.01, ***p < 0.001. versus untreated group.

NPC1 is characterized by neurodegeneration and a hepatosplenic phenotype. To test whether the treatments had any impact on liver and spleen inflammation, *Npc1* ^*−/−*^ mice were treated with metformin, HPβCD, metformin and HPβCD or fed standard chow as a control. The animals were sacrificed at 8 weeks of age and the CD68 expression in the liver and spleen were analysed. The metformin or combined drugs treated *Npc1* ^*−/−*^ animals presented with significantly less CD68^+^ cells in liver and spleen compared to untreated or HPβCD treated *Npc1* ^*−/−*^ animals (Figures 4A,B). Furthermore, we also used quantitative PCR to study the expression profile of proinflammatory cytokine release of *Npc1* ^*−/−*^ mice at 8 weeks of age. The results showed the transcript level of *cd68* and the proinflammatory cytokine (*Tnfα, il1β* and *il6*) was decreased in the metformin or combined drugs treated *Npc1* ^*−/−*^ brain compared to untreated or HPβCD treated *Npc1* ^*−/−*^ animals (Figures 5E–L). In summary, the combined therapy of metformin and HPβCD has a certain protective effect on NPC1 hepatosplenic inflammation.

### Effects of HPβCD And/Or Metformin on Cholesterol Accumulation in Npc1^−/−^ Mice

**FIGURE 6:**
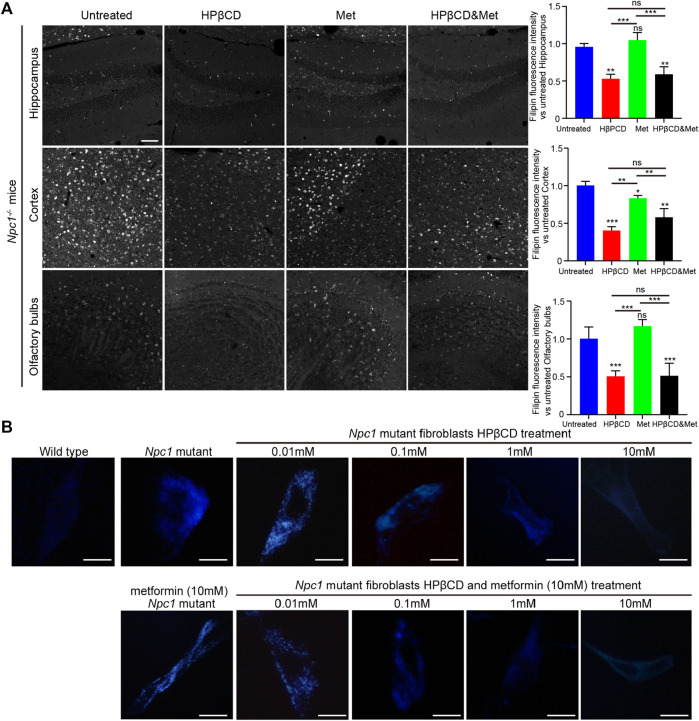
Effect of test drugs on cholesterol accumulation of *Npc1* ^*−/−*^ mice. Filipin staining in the hippocampus, cerebral cortex and olfactory bulb in the indicated groups **(A)**, left) (n = 4) (scale bar: 100 μm). Quantitative analysis of filipin fluorescence intensity using Image J **(A)**, right). Filipin-stained normal and *Npc1* ^*−/−*^ mice fibroblasts after 24 h treatment with HPβCD or/and metformin at various concentrations **(B)** (scale bar: 20 μm). Statistical analysis is based on one-way ANOVA followed by Tukey test, ns represents not significant, *p < 0.05, **p < 0.01,***p < 0.001. All data are provided as means ± SEM.

*Npc1* is a multispan membrane protein that is localized at late endosomes and lysosomes, whereas *Npc2* is a soluble glycoprotein in the lumen. These two proteins bind to cholesterol and cooperate in endosomal cholesterol transport (Qian et al., 2020). One of the symptoms of NPC1 is increased cholesterol storage caused by impaired intracellular cholesterol trafficking. Intracellular cholesterol storage can be assessed using filipin, a cholesterol binding fluorescence dye. Therefore, we test the cholesterol storage in *Npc1* ^*−/−*^ mice that were treated with metformin, HPβCD, metformin and HPβCD or fed standard chow. As expected, the HPβCD treatment can reduced cholesterol storage compared to the untreated *Npc1* ^*−/−*^ mice. However, there are no further reduction of cholesterol accumulation in the metformin or combined drugs treated *Npc1* ^*−/−*^ brain compared to HPβCD treated *Npc1* ^*−/−*^ animals (Figure 6A), thus implying metformin does not affect cholesterol trafficking in NPC1.

To test whether metformin is effective in reducing cholesterol storage *in vitro*, primary *Npc1* ^*−/−*^ mouse fibroblasts were treated with metformin and HPβCD. Increasing concentrations of HPβCD reduced the intracellular fluorescence of filipin. However, metformin did not reduce cholesterol accumulation, either alone or in combination with HPβCD (Figure 6B). These finding may explain why the combination of metformin and HPβCD had no obvious beneficial effect on NPC1 mouse model compared to HPβCD treatment alone.

## Discussion

NPC1 is a highly complex lipid storage disorder that can be targeted with small molecules. Previous studies of monotherapies showed that treatment with 1,000, 2000, or 4,000 mg/kg HPβCD (subcutaneously, once per week) significantly improved survival in *Npc1* ^*−/−*^ mice (Tanaka et al., 2015). We originally chose 4,000 mg/kg every other day for this project because of the plethora of published data defining the NPC1 mouse model at this dose. We found that the mutant mice died after 2–4 injections. Then, we reduced the dose to 2000 mg/kg. The results after HPβCD therapy are in broad agreement with those of previous studies. HPβCD at a dose of 2000 mg/kg could significantly improve the life expectancies and body weights of *Npc1* ^*−/−*^ mice. We refer to several articles about drug treatment in *Npc1* ^*−/−*^ mice. Miglustat was administered to 3 week-old mice, and HPβCD was administered at 6 weeks. We injected the drugs at P20 (nearly 3 weeks old) to ensure the therapeutic effect. Even though HPβCD did not significantly penetrate the blood brain barrier (BBB) on P20, HPβCD lowered the levels of cholesterol in the liver and delayed the onset of neurological signs by 20% (Calias, 2017).

However, the effect of metformin on NPC1 has not been reported. We found that metformin treatment did not change the average lifespan and slightly decreased the body weights of *Npc1* ^*−/−*^ mice. This finding is consistent with previous reports that metformin reduces glycated hemoglobin and fasting plasma glucose while inducing mild weight loss (Stumvoll et al., 1995). Of greater interest were the results of the combination therapies. Anti-inflammatory drugs such as aspirin and ibuprofen are often used in combination with miglustat or HPβCD (Davidson et al., 2009; Smith et al., 2009). Intense research has shown that metformin is a novel treatment against inflammation. In this study, we combined metformin and HPβCD to determine whether dual therapy provides greater functional benefit for *Npc1* ^*−/−*^ mice than monotherapy. We hypothesize that combination treatment with metformin and HPβCD can effectively prolong the survival of *Npc1* ^*−/−*^ mice. Unfortunately, there is no expected superposition effect of the combination therapy for disease treatment. Throughout our study, there were no synergistic increases in survival or weight using metformin plus HPβCD. HPβCD reverses cholesterol accumulation and is one of the very few drugs to do so. However, dual treatment did not further reduce cholesterol storage in mice with NPC1. Our experimental results have preliminarily proven that metformin has no significant beneficial effect on body weight or survival time in HPβCD-treated *Npc1* ^*−/−*^ mice. Most likely, metformin improves inflammation and reduces apoptotic cells in mutant mice due to its anti-inflammatory, antiapoptotic and antioxidative properties through several mechanisms. In this study, we focused on the anti-inflammatory effect of metformin in NPC1. Although cotreatment with metformin did not extend survival time and improve body weights in HPβCD treated *Npc1* ^*−/−*^ mice, it can reduce inflammatory response and inhibited the proinflammatory cytokine release in the brain, liver and spleen of *Npc1* ^*−/−*^ mice.

Disruption of Npc1 function affects lipid transport, lysosomal homeostasis, vesicular trafficking and autophagy. Previous reports suggest that combination therapies may play important roles in the management of NPC1 and potentially exert synergistic effects. Here, we first show that cotreatment with metformin does not extend the survival time of HPβCD-treated *Npc1* ^*−/−*^ mice. However, metformin still plays some role via an anti-inflammatory or other mechanism in NPC1, we would expect a synergistic effect when used in conjunction with HPβCD. In summary, adding metformin treatment to HPβCD treatment in an effort to ameliorate the extended survival time of Npc1 mutant mice was not successful, but it is feasible to reduce the inflammatory response in NPC1.

## Data Availability

The original contributions presented in the study are included in the article, further inquiries can be directed to the corresponding authors.

## References

[B1] Calias P. (2017). 2-Hydroxypropyl-β-cyclodextrins and the Blood-Brain Barrier: Considerations for Niemann-Pick Disease Type C1. Curr. Pharm. Des. 23 (40), 6231–6238. 10.2174/1381612823666171019164220 29065825 PMC5824462

[B2] Cardoso S. Moreira P. I. (2020). Antidiabetic Drugs for Alzheimer's and Parkinson's Diseases: Repurposing Insulin, Metformin, and Thiazolidinediones. Int. Rev. Neurobiol. 155, 37–64. 10.1016/bs.irn.2020.02.010 32854858

[B3] Cologna S. M. Cluzeau C. V. Yanjanin N. M. Blank P. S. Dail M. K. Siebel S. (2014). Human and Mouse Neuroinflammation Markers in Niemann-Pick Disease, Type C1. J. Inherit. Metab. Dis. 37 (1), 83–92. 10.1007/s10545-013-9610-6 23653225 PMC3877698

[B4] Dai S. Dulcey A. E. Hu X. Wassif C. A. Porter F. D. Austin C. P. (2017). Methyl-β-cyclodextrin Restores Impaired Autophagy Flux in Niemann-Pick C1-Deficient Cells through Activation of AMPK. Autophagy 13 (8), 1435–1451. 10.1080/15548627.2017.1329081 28613987 PMC5584846

[B5] Davidson C. D. Ali N. F. Micsenyi M. C. Stephney G. Renault S. Dobrenis K. (2009). Chronic Cyclodextrin Treatment of Murine Niemann-Pick C Disease Ameliorates Neuronal Cholesterol and Glycosphingolipid Storage and Disease Progression. PLoS One 4 (9), e6951. 10.1371/journal.pone.0006951 19750228 PMC2736622

[B6] de la Roche M. Hamilton C. Mortensen R. Jeyaprakash A. A. Ghosh S. Anand P. K. (2018). Trafficking of Cholesterol to the ER Is Required for NLRP3 Inflammasome Activation. J. Cel Biol 217 (10), 3560–3576. 10.1083/jcb.201709057 PMC6168277 30054450

[B7] Demaré S. Kothari A. Calcutt N. A. Fernyhough P. (2021). Metformin as a Potential Therapeutic for Neurological Disease: Mobilizing AMPK to Repair the Nervous System. Expert Rev. Neurother 21 (1), 45–63. 10.1080/14737175.2021.1847645 33161784 PMC9482886

[B8] Gantois I. Popic J. Khoutorsky A. Sonenberg N. (2019). Metformin for Treatment of Fragile X Syndrome and Other Neurological Disorders. Annu. Rev. Med. 70, 167–181. 10.1146/annurev-med-081117-041238 30365357

[B9] Head S. A. Shi W. Q. Yang E. J. Nacev B. A. Hong S. Y. Pasunooti K. K. (2017). Simultaneous Targeting of NPC1 and VDAC1 by Itraconazole Leads to Synergistic Inhibition of mTOR Signaling and Angiogenesis. ACS Chem. Biol. 12 (1), 174–182. 10.1021/acschembio.6b00849 28103683 PMC5791891

[B10] Mahmood K. Naeem M. Rahimnajjad N. A. (2013). Metformin: the Hidden Chronicles of a Magic Drug. Eur. J. Intern. Med. 24 (1), 20–26. 10.1016/j.ejim.2012.10.011 23177353

[B11] Ory D. S. Ottinger E. A. Farhat N. Y. King K. A. Jiang X. Weissfeld L. (2017). Intrathecal 2-Hydroxypropyl-β-Cyclodextrin Decreases Neurological Disease Progression in Niemann-Pick Disease, Type C1: a Non-randomised, Open-Label, Phase 1-2 Trial. Lancet 390 (10104), 1758–1768. 10.1016/S0140-6736(17)31465-4 28803710 PMC6176479

[B12] Ouyang J. Isnard S. Lin J. Fombuena B. Peng X. Chen Y. (2020). GDF-15 as a Weight Watcher for Diabetic and Non-diabetic People Treated with Metformin. Front. Endocrinol. (Lausanne) 11, 581839. 10.3389/fendo.2020.581839 33312159 PMC7708317

[B13] Peake K. B. Vance J. E. (2012). Normalization of Cholesterol Homeostasis by 2-Hydroxypropyl-β-Cyclodextrin in Neurons and Glia from Niemann-Pick C1 (NPC1)-Deficient Mice. J. Biol. Chem. 287 (12), 9290–9298. 10.1074/jbc.M111.326405 22277650 PMC3308731

[B14] Qian H. Wu X. Du X. Yao X. Zhao X. Lee J. (2020). Structural Basis of Low-pH-dependent Lysosomal Cholesterol Egress by NPC1 and NPC2. Cell 182 (1), 98–e18. 10.1016/j.cell.2020.05.020 32544384

[B15] Smith D. Wallom K. L. Williams I. M. Jeyakumar M. Platt F. M. (2009). Beneficial Effects of Anti-inflammatory Therapy in a Mouse Model of Niemann-Pick Disease Type C1. Neurobiol. Dis. 36 (2), 242–251. 10.1016/j.nbd.2009.07.010 19632328

[B16] Stumvoll M. Nurjhan N. Perriello G. Dailey G. Gerich J. E. (1995). Metabolic Effects of Metformin in Non-insulin-dependent Diabetes Mellitus. N. Engl. J. Med. 333, 550–554. 10.1056/NEJM199508313330903 7623903

[B17] Tamura A. Yui N. (2018). Polyrotaxane-based Systemic Delivery of β-cyclodextrins for Potentiating Therapeutic Efficacy in a Mouse Model of Niemann-Pick Type C Disease. J. Control. Release 269, 148–158. 10.1016/j.jconrel.2017.11.016 29138063

[B18] Tanaka Y. Yamada Y. Ishitsuka Y. Matsuo M. Shiraishi K. Wada K. (2015). Efficacy of 2-Hydroxypropyl-β-Cyclodextrin in Niemann-Pick Disease Type C Model Mice and its Pharmacokinetic Analysis in a Patient with the Disease. Biol. Pharm. Bull. 38 (6), 844–851. 10.1248/bpb.b14-00726 26027824

[B19] Vanier M. T. (2015). Complex Lipid Trafficking in Niemann-Pick Disease Type C. J. Inherit. Metab. Dis. 38 (1), 187–199. 10.1007/s10545-014-9794-4 25425283

[B20] Vanier M. T. (2010). Niemann-Pick Disease Type C. Orphanet J. Rare Dis. 5, 16. 10.1186/1750-1172-5-16 20525256 PMC2902432

[B21] Vite C. H. Bagel J. H. Swain G. P. Prociuk M. Sikora T. U. Stein V. M. (2015). Intracisternal Cyclodextrin Prevents Cerebellar Dysfunction and Purkinje Cell Death in Feline Niemann-Pick Type C1 Disease. Sci. Transl Med. 7 (276), 276ra26. 10.1126/scitranslmed.3010101 PMC4415615 25717099

[B22] Williams I. M. Wallom K. L. Smith D. A. Al Eisa N. Smith C. Platt F. M. (2014). Improved Neuroprotection Using Miglustat, Curcumin and Ibuprofen as a Triple Combination Therapy in Niemann-Pick Disease Type C1 Mice. Neurobiol. Dis. 67, 9–17. 10.1016/j.nbd.2014.03.001 24631719

